# Fuzzy Nonlinear Proximal Support Vector Machine for Land Extraction Based on Remote Sensing Image

**DOI:** 10.1371/journal.pone.0069434

**Published:** 2013-07-23

**Authors:** Xiaomei Zhong, Jianping Li, Huacheng Dou, Shijun Deng, Guofei Wang, Yu Jiang, Yongjie Wang, Zebing Zhou, Li Wang, Fei Yan

**Affiliations:** 1 Tianjin Chengjian University, Tianjin, China; 2 Tianjin Institute of Geotechnical Investigation and Surveying, Tianjin, China; 3 Tianjin StarGIS Information Engineering Company Limited, Tianjin, China; 4 Beijing Forestry University, Beijing, China; NASA Jet Propulsion Laboratory, United States of America

## Abstract

Currently, remote sensing technologies were widely employed in the dynamic monitoring of the land. This paper presented an algorithm named fuzzy nonlinear proximal support vector machine (FNPSVM) by basing on ETM^+^ remote sensing image. This algorithm is applied to extract various types of lands of the city Da’an in northern China. Two multi-category strategies, namely “one-against-one” and “one-against-rest” for this algorithm were described in detail and then compared. A fuzzy membership function was presented to reduce the effects of noises or outliers on the data samples. The approaches of feature extraction, feature selection, and several key parameter settings were also given. Numerous experiments were carried out to evaluate its performances including various accuracies (overall accuracies and kappa coefficient), stability, training speed, and classification speed. The FNPSVM classifier was compared to the other three classifiers including the maximum likelihood classifier (MLC), back propagation neural network (BPN), and the proximal support vector machine (PSVM) under different training conditions. The impacts of the selection of training samples, testing samples and features on the four classifiers were also evaluated in these experiments.

## Introduction

Remote sensing (RS) plays a key role in the dynamic monitoring of lands[1]–[3]. Approaches of land extraction that are based on remote sensing image basically include manual visual interpretation and computerized auto-classification. Due to the large number of drawbacks in manual visual interpretation, numerous classification algorithms for computerized auto-classification have been developed; among the most popular are the maximum likelihood classifier, neural network classifiers and decision tree classifiers [4]. The maximum likelihood classifier is a popular classifier on the basis of the assumption that classes in the input data follow a Gaussian distribution. However, there will be errors in the results if the sample data size is not sufficient, where the input data set does not follow the Gaussian distribution and/or the classes have much overlap in their distribution, and therefore resulting in poor separability. The back propagation neural network model is widely applied because of its simplicity and its power to extract useful information from samples [5], [6]. It is a hierarchical design consisting of fully interconnected layers or rows of processing units (with each unit comprising several individual processing elements, which will be explained below). Back propagation belongs to the class of mapping neural network architectures and therefore the information processing function that it carries out is the approximation of a bounded mapping [7]. Furthermore, the approach can effectively avoid some of the problems associated with MLC by simulating the processing patterns of the human brain, although it also has some disadvantages including a slow learning convergent velocity and being easily converging to local minimum [8]. Lastly, the basic idea of decision tree classifier is to break down a complex decision-making process into a collection of simpler decisions, thus providing a solution which is often easier to interpret.

Support vector machine (SVM) is based on statistical learning theory, and aims to determine the location of decision boundaries that produce the optimal separation of classes [9]. This approach, a new classification technique in the field of remote sensing as compared to the above three methods, has quickly gained ground in the past ten years. The SVM classifier can achieve higher accuracies than both the ML (Maximum Likelihood) and ANN (Artificial Neural Network) classifiers [10] can, thus recently it has been applied to classify remote sensing images [11]. Although perfect performance and high classification accuracy can be achieved by basing on the SVM approach, there still are some shortcomings. One of such shortcomings is that the SVM mainly aims at the classification of a small number of training samples, and the cost of calculation increases rapidly with larger data size, especially so for remote sensing data. In order to resolve such issue of high calculation cost, Fung and Mangasarian [12]proposed proximal support vector machine (PSVM), which can also be interpreted as regularized least squares and considered in the much more general context of regularized networks, wherein classifies points are assigned to the closet of two parallel planes that are pushed apart as far as possible. In addition, the method is much more efficient than traditional SVM in terms of running speed because it merely requires the solution of a single system of linear equations. Accuracy and speed of classification are deemed significant in the classification that’s based on remote sensing images. A variety of factors would affect the accuracy and speed of classification: training data size, selection of feature, algorithm parameter setting, just to name a few. Often, real data sets contain noises and the noisy samples might not be representative of a class, as if there is an uncertainty with regard to the class to which they belong. The noises tend to corrupt the data samples, and the optimal hyperplane obtained by the PSVM may be sensitive to noises or outliers in the training sets. As a result, a classifier might not be able to correctly classify some of the data samples having noisy data, so the fuzzy support vector machines [13], [14] and fuzzy linear proximal support machines [15], [16] were proposed to address the problem.

Normally however, real data set is not linearly separable. In this paper, we proposed the fuzzy nonlinear proximal support vector machine (FNPSVM) to extract different types of lands, and this technique is actually a fuzzy non-linear extension of the existing PSVM methods. In addition, we defined a fuzzy membership function that assigned a fuzzy membership to each data point, such that different data points could have different effects in the learning of the separating hyperplane. Additionally, for the purpose of improving algorithm performance, we presented the approaches of some key parameters of this algorithm, as well as the approaches of feature extraction and feature selection. And lastly, we compared our algorithm with the other three classifiers (MLC, BPN, and PSVM).

The paper is organized as follows:

Section 2 discusses in detail the architectures of PSVM and FNPSVM.

Training algorithm of FNPSVM is shown in section 3.

Experimental results of the algorithm and discussion are presented in section 4.

Section 5 contains the concluding remarks.

## Architectures of PSVM and FNPSVM

### Architecture of PSVM

**Figure 1:**
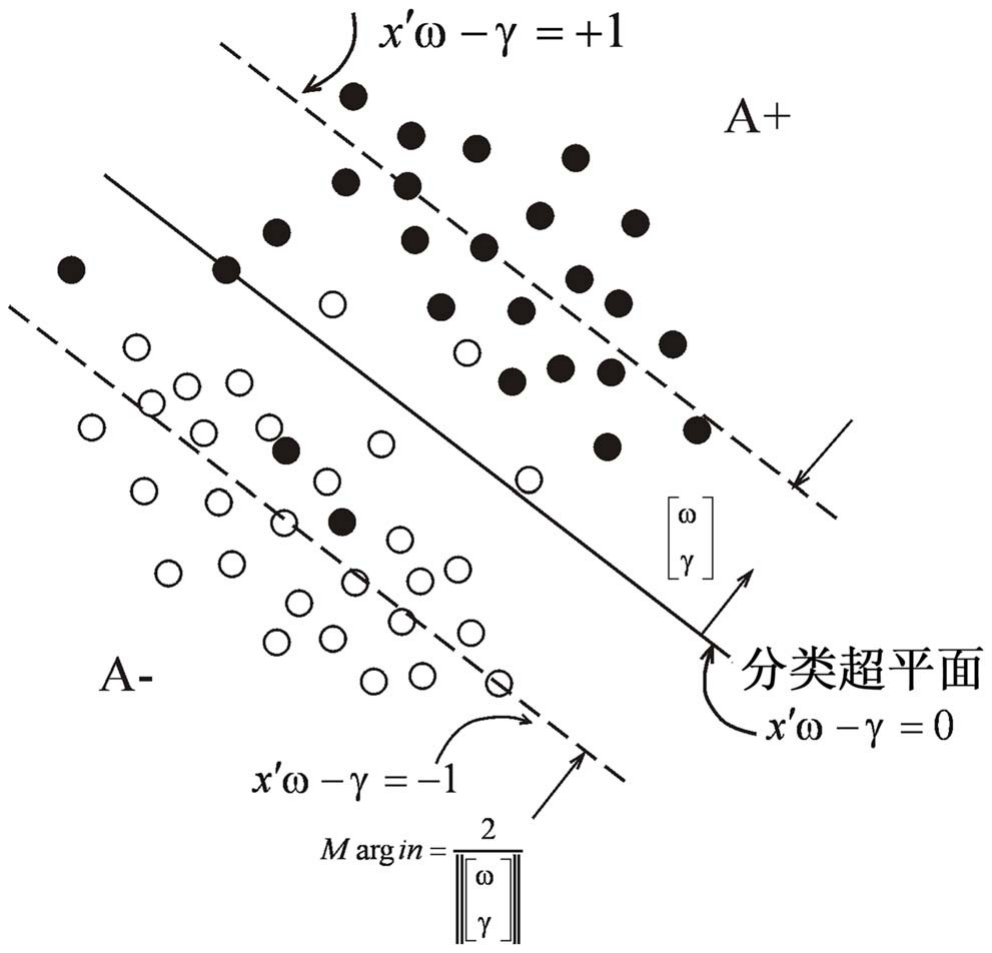
The Proximal Support Vector Machine Classifier: The planes 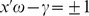 around which points of the sets A+ and A- cluster and which are pushed apart by the optimization problem (1).



To deduce our FNPSVM algorithm, we briefly introduce the binary category proximal support vector machine first. Let the data set consisting of *m* points in the *n*-dimensional real space 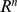 be represented by the 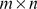 matrix, and let each point be represented by an *n*-dimensional row eigenvector 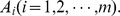 In the case of binary classification, each data point 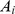 in the class of *A*+ or *A*- is specified by a given 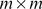 diagonal matrix *D*, with +1 or -1 elements along its diagonal. The target is separating the *m* data points into *A*+ and *A*-, as depicted in Figure 1. For the problem, the proximal support vector machine with a linear kernel [12] is given by the following quadratic program with parameter *c*>0 (which controls the tradeoff between the margin and the error) and linear equality constraint:

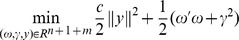


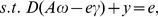
(1)where *e* is an *m*-dimensional vector of ones, and *y* is an error vector. When the two classes are strictly linearly separable, 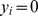 in (1) (which is not the case shown in Figure 1). As depicted in Figure 1, the variables 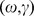 determine the orientation and location of the proximal planes:



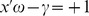


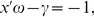
(2)around which the points of each class are clustered and which are pushed apart as far as possible by the term 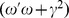 in the objective function. Consequently, the plane:
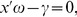
(3)midway between and parallel to the proximal planes (2), is a separating plane that approximately separates A+ from A- as depicted in Figure 1. The distance 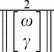 is called the “margin” (see Figure 1), and maximizing the margin enhances the generalization capability of a support vector machine [9], [17]. The approximate separating plane (3) shown in Figure 1, acts as decision function as follows:
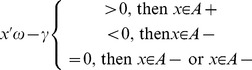
(4)


### Architecture of FNPSVM

In this paper, we will employ the following norms of a vector 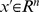 [17]:
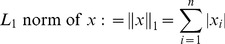
(5)

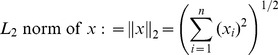
(6)

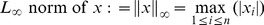
(7)


#### The fuzzy nonlinear binary category proximal support vector machine

Generally, real data sets are corrupted with noises. And as a result, it’s not always the case that one classifier obtained by training with noisy data would correctly classify some of the data samples. Since the optimal hyperplane only depends on a small part of the data points, it may become sensitive to noises or outliers in the training set [18], [19]. We can associate each data point with a fuzzy membership that reflects their relative degrees as meaningful data, and accounts for the uncertainty in the class to which it belongs. Those noises or outliers are treated as less important and have lower fuzzy membership. This equips the classifier with the ability to train data that has noises or outliers. Such is done by setting lower fuzzy memberships to the data points that are considered to be noises or outliers with higher probability. A classifier that is able to use information regarding this fuzzy degree can improve its performance, and reduce the effects of noise or outliers. Thus we proposed the following the optimization problem in determining the classifier:
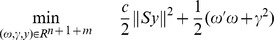


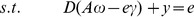
(8)where 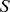 denotes a diagonal matrix, i.e. 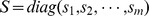, whose diagonal elements correspond to the membership values of the data samples belonging to A+ or A-; and *e* is the vector of plus ones. And 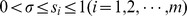.

According to the objective function of (8), *y* can be replaced by 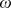 and 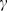, so we then arrive at the following unconstrained minimization problem:
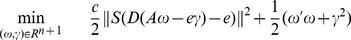
(9)To obtain fuzzy nonlinear proximal classifier, we modify formula (9) as in [12], [20] first by substituting the variable 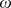 with its dual equivalent 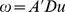, and then by modifying the last term of the objective function to be the norm of the new dual variable 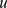 and 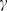. Now we obtain the following problem:
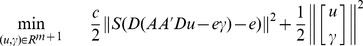
(10)If we now replace the linear kernel AA’ by a nonlinear kernel K (A, A)’, we obtain:



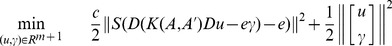
(11)Let 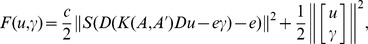 and setting one-order derivative of 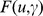 with respect to 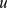 and 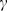 to zero, i.e. 
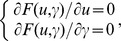
 we arrive at the following formula:
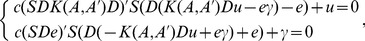
(12)where both *D* and *S* are diagonal matrices, and so that 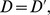 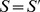 and 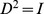. Further, we deal with the above formula (12), and obtain the equations with respect to 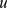 and 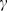:
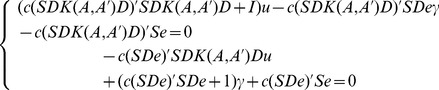
(13)Now let



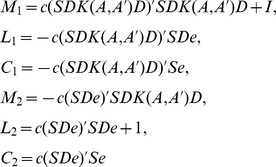
And thus formula (13) can be expressed by the following formula:
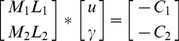
(14)We can work out 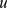 and 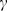 by solving formula (14), and hence the binary category nonlinear classifier can be written as follows:
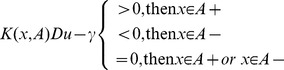
(15)


#### The fuzzy nonlinear proximal support vector machine

There are roughly four types of support vector machines that handle multi-class problems [21]. Two strategies have been proposed to adapt the SVM to N-class problems [22], namely the “one-against-one” strategy and the “one-against-rest” strategy. The “one-against-one” strategy is to construct a machine for each pair of classes, resulting in N (N­ 1)/2 machines. When applied to a test pixel, each machine gives one vote to the winning class, and the pixel is labeled with the class having most votes. The “one-against-rest” strategy is to break the N-class case into N two-class cases, in each of which a machine is trained to classify one class against all others [4]. In this paper, we employed the above mentioned strategies.

♦“One-against-one” strategy:



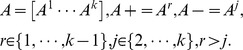
Here, *k* is the class number, while 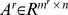 and 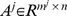 represent the 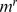 and 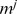 points in class *r* and class *j*, respectively. Let 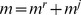, and thus *D* is a 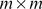 diagonal matrix as follows:



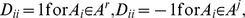
From formula (14), the 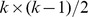 unique 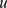 and 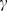 can be obtained, and thus 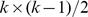 proximal surfaces are generated:



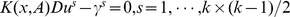
A new given point 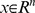 is assigned the *i* ^th^ class 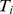 (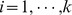) timed by 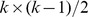 proximal surfaces, and finally 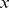 is assigned *i* ^th^ class in terms of the following formula:



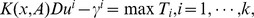
supposing the dataset is to be classified into M classes. Therefore, M binary SVM classifiers may be created where each classifier is trained to distinguish one class from the remaining M-1 classes. For example, class one binary classifier is designed to discriminate between class one data vectors and the data vectors of the remaining classes. Other SVM classifiers are constructed in the same manner. During the testing or application phase, data vectors are classified by finding margin from the linear separating hyperplane. The final output is the class that corresponds to the SVM with the largest margin [23].

♦“One-against-rest” strategy:



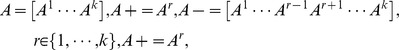
where *k* is the class number, 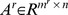 represents the 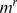 points in class *r*. Letting 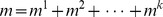, so that *D* is a 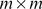 diagonal matrix as follows:



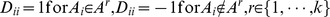
From formula (14), the *k* unique 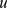 and 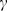 can be obtained, and thus *k* proximal surfaces are generated:



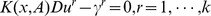
A new given point 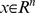 is assigned class *t,* depending on which of the *k* nonlinear halfspaces generated by the *k* surfaces it lies deepest in, namely:



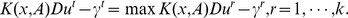
In this method, SVM classifiers for all possible pairs of classes are created. Therefore, for M classes, there will be binary classifiers. The output from each classifier in the form of a class label is obtained. The class label that occurs most is assigned to that point in the data vector. In case of a tie, a tie-breaking strategy may be adopted. A common tie-breaking strategy is to randomly select one of the class labels that are tied [23].

## Training algorithm of FNPSVM

### Fuzzy membership model

**Figure 2:**
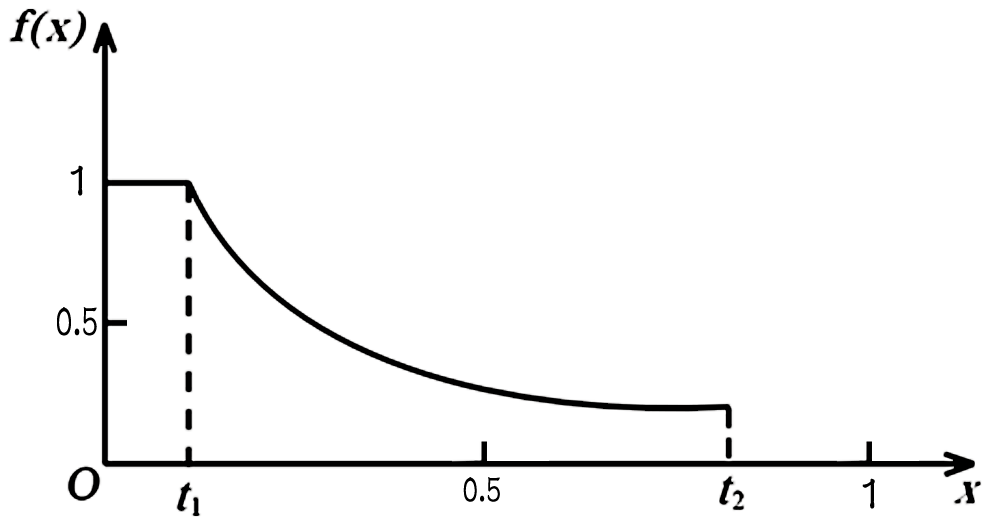
Figure of Fuzzy Membership Function: *t*_1_ and *t*_2_ that tune the fuzzy membership of each data point in the training are two user-defined constants, and they determine the range in which the data sample absolutely does or does not belong to a given class.

In order to improve classification performance and to reduce the corruption of data samples from noises, we defined a fuzzy membership function to a given class, where a membership is assigned to each data point. It is written as:
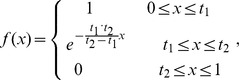
where *x* denotes the distance between the data sample and the center of the class that it belongs to. In addition, *t*_1_ and *t*_2_ that tune the fuzzy membership of each data point in the training are two user-defined constants, and they determine the range in which the data sample absolutely does or does not belong to a given class. On the other hand, they also control the figure of the curve (see Figure 2).

A reducing value of x would indicate that the distance between the data sample point and the center of the given class is smaller, and the probability of this sample belonging to this certain class is higher. When x is between 0 and *t*_1_, the data sample point belongs to the given class with absolute certainty; and when x is between *t*_2_ and 1, the data sample point doesn’t belong to the given class. When the value of x is known, the values of *t*_1_ and *t*_2_ would influence the values of fuzzy memberships, and thus would also influence the ultimate classification result.

The distance *x* is the key of each training sample’s fuzzy membership, and it can be obtained as follows:
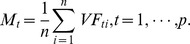


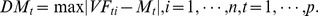


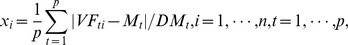
where *n* is the number of training samples to a given class, and *p* is the number of feature selected, with 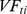 representing the *t* ^th^ feature value of the *i* ^th^ sample. 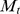 is the mean value of *t* ^th^ feature of *n* samples to a given class; 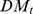 is the max value of the distances between all sample points and the center (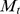) of the *t* ^th^ feature to a given class; and 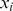 denotes the average distance between the *i* ^th^ sample and the centers of all features.

### Sample Selection

The choice in sample size and sampling design affect the performance and reliability of a classifier. Sufficient samples are necessary. A previous study indicated that this factor alone could be more important than the selection of classification algorithms in obtaining accurate classifications [24].

Sample selection includes two parts, namely sample data size and selection method. Increases in sample data size generally will lead to improved performances, though at the same time resulting in a higher calculation cost. The sample size must be sufficient enough to provide a representatively meaningful basis for training of a classifier and for accuracy assessment. The basic sampling designs, such as simple random sampling, can be appropriate if the sample size is large [25] enough. The adoption of a simple sampling design is also valuable in helping to meet the requirements of a broad range of users [26]. In this paper, we apply simple random sampling design to collect training samples and testing samples.

### Kernel Function Strategy

The concept of the kernel is introduced to extend SVM’s ability in dealing with nonlinear classification. It can transform non-linear boundaries in low-dimensional space into linear ones in high-dimensional space by mapping feature vector into a high-dimensional space, and thus the training data can be classified in the high-dimensional space without knowing the specific form of the mapping function. A kernel function is a generalization of the distance metric that measures the distance between two data points as the data points are mapped into a high dimensional space in which the data are more clearly separable [27], [28].

Three kernel functions for nonlinear SVM, including the radial basis function (RBF), the polynomial, and the sigmoid are widely used. In this paper, we have adopted the Gaussian RBF kernel as the default kernel function model due to the fact that: (1) The RBF kernel can handle the case where the relation between class labels and attributes is nonlinear [29]; (2) The polynomial function spends a longer time in the training stage of SVM, and some previous studies [30]–[32] have reported that the RBF function would provide better performance compared to polynomial function. In addition, the polynomial kernel has more hyper parameters than RBF kernel does, and may approach infinity or zero while the degree is large [29]; (3) The sigmoid kernel behaves like the RBF under certain parameters; however, it is not valid under some parameters [9]; (4) When the size of sample data is quite large, convergent ability of RBF kernel is stronger than that of the other kernels above.

The Gaussian kernel function is expressed as:
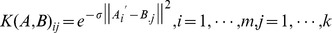
Here, the matrix 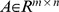, and 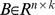;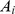 is the *i* ^th^ row of 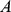, which is a row vector in 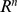, while 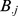 is the *j* ^th^ column of 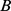; the kernel 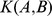 maps 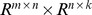 into 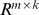. In particular, if *x* and *y* are column vectors in 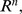 then 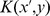 is a real number, 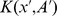 is a row vector in 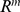, and 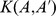 is a 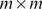 matrix. The parameter 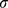 of the RBF kernel is a user-defined positive constant regulating the width of the Gaussian kernel, which has an important impact on kernel performance. There is however little guidance in the literatures on the criteria of selecting kernel-specific parameters [33], hence we carried out lots of trials to acquire the optimal parameter 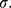


### Parameter Selection Method

Regardless of using a simple or a more complex classifier, the learning parameters have to be chosen carefully in order to yield a good classification performance. The FNPSVM algorithm proposed in this paper requires four given parameters, specifically *c*, 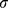, *t*_1_ and *t*_2_. Vapnik [9] discovered that varying kernel functions would slightly affect classification results of SVM, while the parameters of the kernel functions and penalty constant *c* would have a strong effect on the performance of SVM.

One such parameter *c*>0 is an important quantity in determining a trade-off between the empirical error (number of wrongly classified inputs) and the complexity of the found solution. Normally large values for *c* lead to fewer training errors (and a narrower margin), all at the cost of more training time; whereas small values generate a larger margin, with more errors and more training points situated inside the margin. Since the number of training errors cannot be interpreted as an estimate of the true risk, this knowledge does not really help in choosing a suitable value for the parameter. The parameter 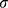 of the Gaussian kernel affects the complexity of the decision boundary. Improper selection of these two parameters can cause over-fitting or under-fitting problems [29], [34]. Nevertheless, there is little explicit guidance to solve the problem of choosing parameters for SVM. Recently, Hsu [35] suggested a method in determining parameters, namely grid-search and cross validation. For multi-category however, the cross validation method is not feasible. In this paper, we advanced his method and proposed an approach named the multi-layer grid search and random-validation.

The basic idea of random-validation is that we randomly divide the sample set into training set and test set of different size to each category. The test set is sequentially tested using the classifier trained on the training set, and the classification accuracy is derived. The above procedure is iteratively executed for *n* times during each cycle, and *n* accuracies are obtained. Finally, the random-validation accuracy is the mean of *n* accuracies.

We recommend the “multi-layer grid search” method on *c* and 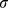 using *n* random-validation, in order to accurately find the optimal parameters while lowering computational cost. We first acquire the boundary of the parameters c and 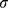, and the 2-dimentional grid of pairs of 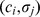 is roughly constructed. Here, 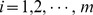, and 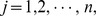 thus 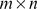 gird-plane and 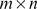 pairs of 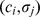 are obtained. The FNPSVM algorithm uses each pair of 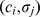 to learn by basing on n random-validation, and obtains the classification accuracy. The corresponding 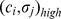 of the best accuracy is the optimal pair. If the best accuracy does not satisfy the requirement of classification, a new 2-dimentional grid-plane that’s based on the center of the pair of 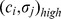 should be constructed, and the learning by using new pairs of 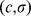 in the new grid-plane is executed to acquire higher accuracy. The above procedure is performed iteratively to find the optimal parameters *c* and 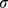.

Although the multi-layer grid search and random-validation seem simple, it is actually practical because of the fact that: (1) For each parameter, a finite number of possible values is prescribed, and then all possible combinations of 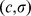 are considered to find one that yields the best result; (2) the computational time in finding good parameters through the approach isn’t much more than that of advanced methods, since there are only two parameters (generally the complexity of grid search grows exponentially with the number of parameter); (3) The grid-search can be easily parallelized because each 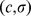 is independent, unlike some other advanced methods that require iterative processes.

## Experiments and Discussion

All experiments were run on 1800 MHz ADM Sempron (tm) processor 3000^+^ under Windows XP using Matlab 7.0 compiler. We have adopted the classification criterion of Chen [36]; saline-alkalized lands are classified into heavy saline-alkalized land, moderate saline-alkalized land, and light saline-alkalized land.

### Classification Experiments Using ETM^+^ Image

#### Experiment summary

We have selected Da’an, a city in northern China with a total area of 4,879 km^2^ as our test area. Multi-spectral (Landsat-7 ETM^+^) remote sensing data (30 m spatial resolution, UTM project) acquired on August 30^th^, 2000 was used to classify the image data into nine land cover types (heavy saline-alkalized land, moderate saline-alkalized land, light saline-alkalized land, water area, cropland, grassland, rural residential area, urban residential area, and sand land).

According to the topographic maps of Da’an city (1∶100,000 scale), we implemented precise geometric correction and resampling of the image. Geometric correction of image was accomplished through two-order polynomial while resampling was achieved through cubic convolution with the error of matching less than one pixel. We selected 270 samples (90 for training and 180 for testing) for each class using a random sampling procedure from the image, totally 810 training samples and 1,620 test samples for nine classes. For each sample set, the test set was independent of the training set.

To demonstrate the effectiveness of the proposed method, both “one-against-one” and “one-against-rest” strategies that are based on Gaussian RBF kernel in dealing with the *n*-class case were used, and the results (various accuracies, training speed, and classification speed) obtained using FNPSVM algorithm were compared with those derived from the four conventional classification methods including the maximum likelihood classifier (MLC), back propagation neural network (BPN), support vector machine (SVM), and proximal support vector machine (PSVM) under different training conditions (shown in Table 1).

**Table 1:** Training data conditions under which the classification algorithms were tested.

Sample size		Number of features	Training case no.
Training sample number	Testing sample number		
60	210	4	A
		7	B
		10	C
		14	D
90	180	4	E
		7	F
		10	G
		14	H
120	150	4	I
		7	J
		10	K
		14	L

#### Feature extraction and feature selection

(1) Feature extraction. Feature extraction has a strong impact on classification accuracy. In this paper, we extracted 14 features, including six bands of ETM^+^ image, the first principle components of K-L transform and K-T transform, soil index, NDVI (normalized difference vegetation index), composition index, as well as *H* (hue), *S* (saturation), and *I* (intensity) color components of *HSI* color space. Some of the features can be obtained as follows:
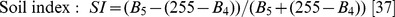


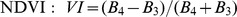


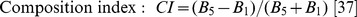
Here 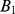, 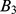, 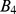, 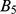 represent the first band, third band, forth band, and fifth band of ETM^+^ image, respectively.

In the field of digital image processing, a number of color models were proposed, such as *RGB*, *HSI*, *CIE*, etc. But selecting the most optimal color space is still a problem in color image segmentation [20].

The *RGB* color model is suitable for color display, but less so for color analysis because of its high correlation among *R*, *G*, and *B* color components [38]. In color image processing and analysis, we know that: (1) *H* and *S* components are closely correlated to the color sense of the eyes; (2) Hue information and intensity information are distinctly differentiated in *HSI* model; (3) By HSI model, computer program can easily process color information after the color sense of the eye has been transformed into specific values, so we extracted *H*, *S*, and *I* color components of *HSI* color space as three features of classification. False color image composite of bands 5, 4, and 2 were performed, after which the image was exported into *RGB* image. And finally the *RGB* model was transformed into *HSI* model according to the following formulas [39]:
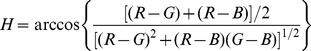


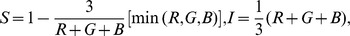
(2) Feature selection.Normally, the size of a real dataset is so large that learning might not work, and the running time of a learning algorithm might be drastically increased before removing these unwanted features. Thus we must select some features that are neither irrelevant nor redundant to the target concept.

Feature selection for classification is a well-researched problem, striving to improve the classifier’s generalization ability, and to reduce the dimensionality and the computational complexity. It directly reduces the number of original features by selecting a subset of them that still retains sufficient information for classification [40]. Feature selection attempts to select the minimally sized subset of features according to the following criterion. The criterion can be [41]:

1. The classification accuracy does not significantly decrease; and
2. The resulting class distribution when given only the values for the selected features, is as close as possible to the original class distribution when given all features.

For this paper, in terms of the above criterion, the data types and the characteristics of remote sensing image, we adopted traditional DB Index rules which used the methods of between-class scatter and within-class scatter to select classification features. DB Index rules are as follows [42]:

1) 
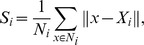
where 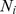 denotes the number of samples of *i* ^th^ class; and 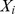 represents the center of the *i* ^th^ class.

2) 
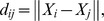
where 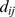 is the distance between the centers of the two classes.

3) DB Index 
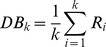
, 
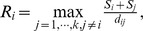
where *k* is the number of classes.

The smaller the value of 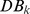 is, the better the performance of classification is. Based on the above rules and 270 sample points of each category, we obtained DB indices of fourteen features and their ranks (see Table 2).

**Table 2:** DB indices of fourteen features and their ranks.

Rank	Feature	DB index
1	the 6^th^ band of ETM image	2.0408
2	the 5^th^ band of ETM image	4.2657
3	the 4^th^ band of ETM image	5.0092
4	CI (Composition Index)	6.3319
5	the 1^st^ component of K-L transform	7.0428
6	*H* component of *HSI* color space	7.6135
7	SI (Soil Index)	8.5819
8	NDVI (Normalized Difference Vegetation Index)	9.8511
9	the 1^st^ component of K-T transform	10.8020
10	the 1^st^ band of ETM image	14.8599
11	the 3^rd^ band of ETM image	25.2807
12	the 2^nd^ band of ETM image	26.7408
13	*I* component of *HIS* color space	29.8844
14	*S* component of *HIS* color space	153.2745

#### Parameter setting

Due to the differing nature of the impacts that algorithm parameters have on different algorithms, it is impossible to account for such differences in evaluating the comparative performances of the algorithms [4]. To avoid this problem, the corresponding parameters of the best performance of each algorithm were chosen for the purpose of comparison.

(1) Parameter setting of PSVM and FNPSVM.The performance of classification algorithms is affected by the parameter settings of those algorithms. As described in section 3.4, we searched for the optimal parameters *t*_1_, *t*_2_, *c*, and 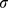 for FNPSVM classifier. In this procedure, we used two steps to find the best parameters. In the first step, we set the parameters *t*_1_ = 0.1 and *t*_2_ = 0.8, and searched for the kernel parameter 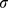 and penalty constant *c* as described in section 3.5. In the second step, we set the parameters 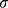 and *c* as found in the first step, and searched for the parameters *t*_1_ and *t*_2_ of the fuzzy membership mapping function. In the first step, we constructed the two-dimensional grid for the first layer. The values of *c* and 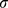 were prescribed from 2^−14^ to 2^14^, multiplied by 2^4^. The grid-search using 5-time random-validation was executed, and we found that the optimal parameter pair 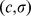 was (2^10^, 2^−10^), having the highest overall classification accuracy (93.31%) and kappa value (0.9248). Table 3 summarized the results of first-layer grid-search. Subsequently we constructed the second-layer grid based on the center (2^10^, 2^−10^); and the values of *c* and 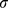 were chosen from 2^7^ to 2^13^ and from 2^−7^ to 2^−13^, multiplied by 2 respectively; and the grid-search using 5-time random-validation was implemented. As was shown in Table 4, *c* = 2^13^ and 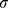 = 2^−13^ gave the best overall classification accuracy (93.56%) and kappa coefficient (0.9275). As the accuracies could fundamentally satisfy our classification demand, we began the next step, where we set the parameters *c*_ = _2^13^ and 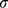 = 2^−13^, and searched for the parameters *t*_1_ and *t*_2_. Unfortunately, we couldn’t find that the changes of parameters *t*_1_ (0.05∼0.2) and *t*_2_ (0.7∼0.9) to be able to significantly improve the performance of the FNPSVM, hence we set *t*_1_ = 0.1, *t*_2_ = 0.8.

**Table 3:** The overall accuracies (%) and kappa coefficients of the first layer grid-search using 5-time random-validation based on ETM^+^ image.

*c* σ	2^−14^	2^−10^	2^−6^	2^−2^	2^2^	2^6^	2^10^	2^14^
2^−14^	40.87/0.3348	69.15/0.6530	17.92/0.0766	11.11/0	11.11/0	11.08/0	11.11/0	11.11/0
2^−10^	59.65/0.5461	74.25/0.7103	59.18/0.5408	12.45/0.0151	11.09/0	11.11/0	11.11/0	11.11/0
2^−6^	64.00/0.5950	81.48/0.7916	75.83/0.7281	25.00/0.1563	11.43/0.0036	11.52/0.0046	11.34/0.0026	11.34/0.0026
2^−2^	76.93/0.7405	88.02/0.8653	85.30/0.8346	42.13/0.3490	12.53/0.0160	11.57/0.0051	11.62/0.0058	11.49/0.0043
2^2^	85.60/0.8380	90.71/0.8955	90.32/0.8911	47.95/0.4145	13.15/0.0230	11.55/0.0050	11.60/0.0055	11.54/0.0048
2^6^	89.33/0.8800	92.78/0.9188	89.91/0.8865	46.20/0.3948	13.80/0.0303	11.42/0.0035	11.43/0.0036	11.61/0.0056
2^10^	91.86/0.9085	93.31/0.9248	89.36/0.8803	48.60/0.4218	13.49/0.0268	11.57/0.0051	11.55/0.0050	11.70/0.0066
2^14^	92.44/0.9150	93.08/0.9221	87.70/0.8616	45.14/0.3828	13.79/0.0301	11.71/0.0068	11.61/0.0056	11.67/0.0063

**Table 4:** The overall accuracies (%) and kappa coefficients of the second layer grid-search using 5-time random-validation based on ETM^+^ image.

*c σ*	2^−7^	2^−8^	2^−9^	2^−10^	2^−11^	2^−12^	2^−13^
2^7^	91.20/0.9010	92.82/0.9193	92.34/0.9138	92.91/0.9203	92.71/0.9180	92.11/0.9113	91.00/0.8988
2^8^	91.14/0.9003	92.45/0.9151	92.74/0.9183	92.71/0.9180	92.42/0.9148	92.07/0.9108	91.71/0.9068
2^9^	91.34/0.9026	91.95/0.9095	92.94/0.9206	92.68/0.9176	92.75/0.9185	92.57/0.9165	92.10/0.9111
2^10^	89.70/0.8841	92.45/0.9151	93.36/0.9253	92.48/0.9155	93.17/0.9231	92.91/0.9203	92.08/0.9110
2^11^	90.99/0.8986	91.37/0.9030	92.37/0.9141	92.75/0.9185	92.99/0.9211	92.29/0.9133	92.57/0.9165
2^12^	90.19/0.8896	91.49/0.9043	92.23/0.9126	93.08/0.9221	92.63/0.9171	92.96/0.9208	93.06/0.9220
2^13^	90.16/0.8893	91.57/0.9051	92.42/0.9148	92.82/0.9193	93.05/0.9218	92.80/0.9190	93.56/0.9275

(2) Parameter setting of BP neural network.There are many parameters associated with BP neural network, including neuron number, transfer function, learning rate, iteration time and so on. It is not easy to know beforehand which values of these parameters are the best for a problem. Consequently in this paper, in order to yield the optimal classification performances, the settings of some key parameters of BP neural network were achieved by repeated trials and some experiences from previous studying.

A BP neural network with a hidden layer can approximate with arbitrary precision an arbitrary non-linear function that’s defined on a compact set of *R* ^n^ [43], [44].We employed three-layer BP neural network including input layer, hidden layer and output layer. The number of neurons in the hidden layer is one of the primary parameters of BPN algorithm; currently however there is no authoritative rule to determine it. Larger number of hidden units leads to a poor generalization and increases training time, but too few neurons would cause the networks to unfit the training set and to prevent the correct mapping of inputs and outputs. In this paper, the number of neurons in the hidden layer was determined by the empirical formula [44] to be 20, thus the network structure became *n*-20-9 (*n* denotes the number of features).

**Figure 3:**
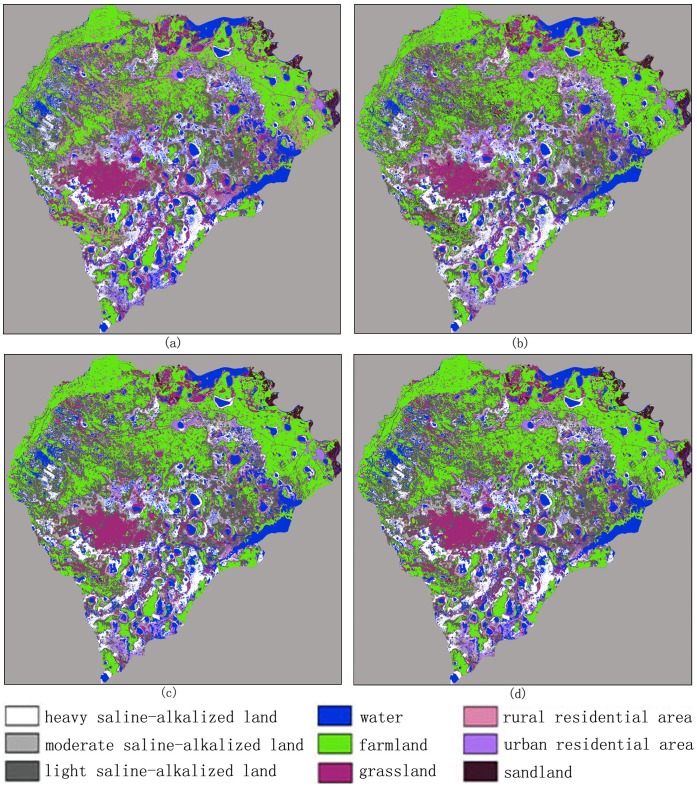
Classification maps for the test area in northern China using various classifiers under the same training case (90 training samples for each class, 10 features). (a) MLC algorithm. (b) BPN algorithm, 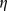 = 0.5, 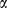 = 0.8, 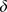 = 10–20, 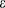 = 10–6. (c) PSVM algorithm, c = 213, 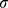 = 2–13. (d) FNPSVM, t1 = 0.1, t2 = 0.8, c = 213 and 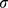 = 2–13.

We chose log-sigmoid function as the transfer functions from input layer, while setting the limit on the neural network’s iteration number to be 1,000 times for each desired output. Levenberg-Marquard optimum algorithm (trainlm function in Matlab software) was utilized as the training function because it could greatly increase the training speed of the network by utilizing a lot of memory. Gradient descent with momentum weight and bias learning function was employed to calculate a given neuron’s weight change from the neuron’s input and error, the weight, learning rate, and the momentum constant according to the gradient descent with momentum. The other parameters of the network are chosen as follows: learning rate 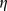 = 0.5, momentum factor 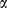 = 0.8, minimum gradient 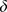 = 10^−20^, and minimum mean square error 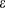 = 10^−6^. Figure 3 shows the classification maps using the MLC, BPN, PSVM, and FNPSVM, all based on the settings of above parameters of various classifiers.

#### Performance assessments

**Figure 4:**
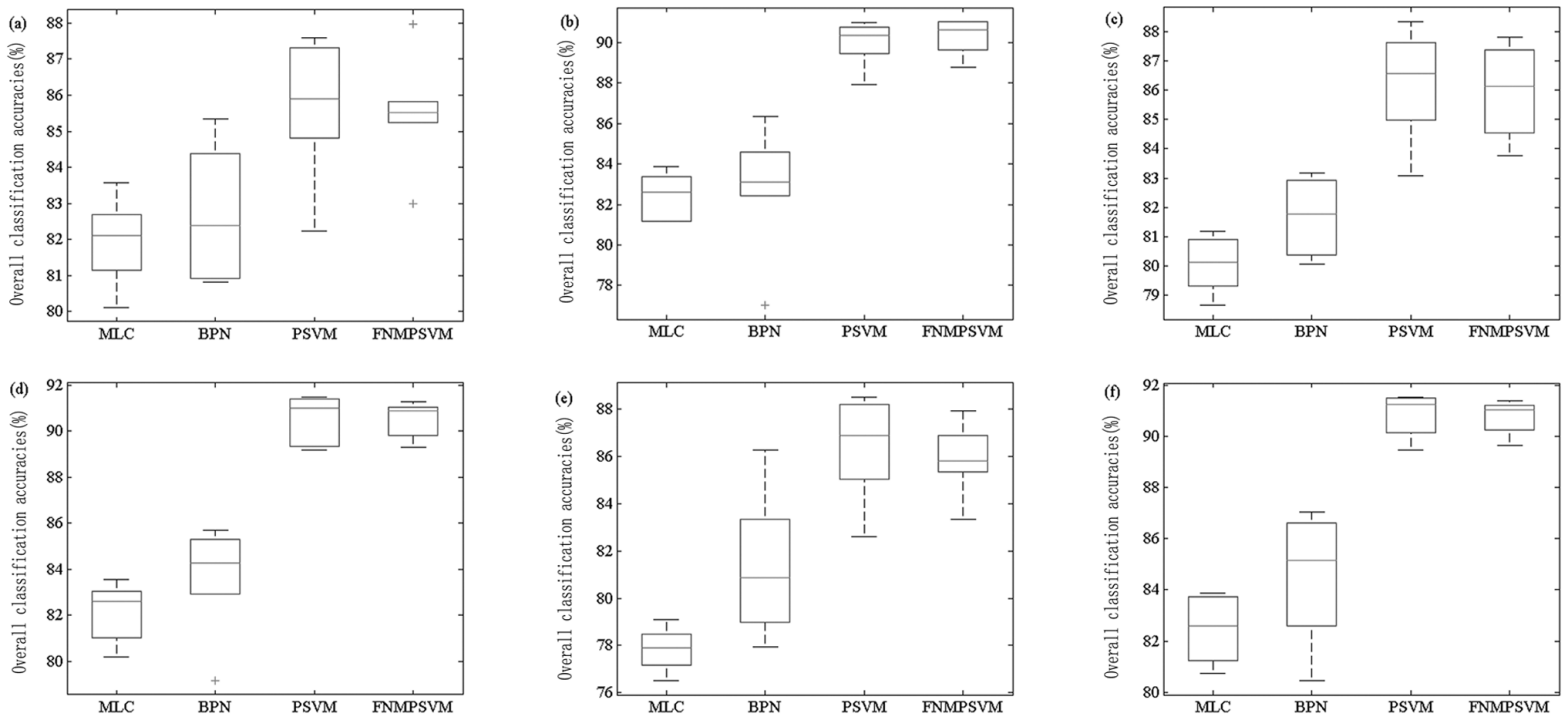
Boxplots of the overall classification accuracies developed by randomly selecting training samples and testing samples for six times from 270 samples of each class based on ETM^+^ image. (a) Training samples = 60, testing samples = 210, number of features = 4. (b) Training samples = 60, testing samples = 210, number of features = 10. (c) Training samples = 90, testing samples = 180, number of features = 4. (d) Training samples = 90, testing samples = 180, number of features = 10. (e) Training samples = 120, testing samples = 150, number of features = 4. (f) Training samples = 120, testing samples = 150, number of features = 10.

Normally, settings of the various parameters on different algorithms affect the classification results, so it is difficult to evaluate the comparative performances of the algorithms because of the changing parameters. To address this problem, the best performance of each algorithm on each training case was listed in the following tables. The criterion for evaluating the performances of classification algorithms includes accuracy, speed, stability and comprehensibility, among others [4]. In this paper, we chose one group of criteria, consisting of classification accuracy, speed and stability to assess the performances of different algorithms. Table 5 gave overall accuracies and kappa coefficients using various multi-class strategies and classifiers with ETM^+^ data on different cases. Using different classifiers under different training conditions, Table 6 gave training speed and classification speed of the entire data set. Means and standard deviations of the overall classification accuracies basing on different training samples, testing samples and features, were manifested in Table 7. Figure 4 shows the boxplots of the overall classification accuracies, developed by randomly selecting training samples and testing samples from the 270 samples of each class for six times.

**Table 5:** Overall accuracies (%) and kappa coefficients using various multi-class strategies and classifiers on different cases based on ETM^+^ image.

Trainingcase no.	MLC	BPN	PSVM		FNPSVM	
	OA/KC	OA/KC	One against oneOA/KC	One against restOA/KC	One against oneOA/KC	One against restOA/KC
A	81.28/0.7419	81.94/0.7923	87.82/0.8601	85.59/0.8347	87.97/0.8617	85.45/0.8332
B	81.33/0.7427	85.05/0.8291	89.36/0.8777	88.21/0.8645	89.23/0.8763	88.02/0.8624
C	82.34/0.7548	85.09/0.8288	91.19/0.8989	89.05/0.8739	91.14/0.8982	88.59/0.8687
D	80.15/0.7291	82.66/0.8006	91.20/0.8989	90.21/0.8875	91.21/0.8989	89.57/0.8802
E	81.85/0.7484	82.93/0.8034	87.52/0.8566	86.16/0.8411	87.87/0.8606	86.09/0.8405
F	82.35/0.7541	86.29/0.8430	89.84/0.8831	88.24/0.8648	89.88/0.8836	88.29/0.8655
G	84.18/0.7755	84.83/0.8258	91.05/0.8985	88.02/0.8635	91.92/0.9071	88.87/0.8719
H	81.41/0.7433	83.03/0.8048	91.85/0.9062	90.26/0.8880	92.81/0.9158	90.93/0.8943
I	82.02/0.7503	82.05/0.7935	87.76/0.8593	86.50/0.8449	88.00/0.8621	86.51/0.8451
J	82.39/0.7547	88.56/0.8685	89.91/0.8840	88.83/0.8715	90.73/0.8919	89.79/0.8811
K	84.85/0.7831	86.47/0.8442	91.99/0.9079	89.16/0.8751	91.71/0.9046	88.79/0.8709
L	81.72/0.7460	84.01/0.8157	92.12/0.9093	90.15/0.8868	92.94/0.9172	90.83/0.8931

*Note*: OA and KC denote overall accuracy (%) and kappa coefficient, respectively.

**Table 6:** Training time and classification time of whole data set (4,037,099 pixels) using various classifiers on different cases based on ETM^+^ image unit:second.

Training condition	MLC		BPN		PSVM		FNPSVM	
	Training time	Classification time	Training time	Classification time	Trainingtime	Classification time	Training time	Classification time
Training samples = 1080 Feature number = 4	13	408	556	775	54	62925	17	27968
Training samples = 1080 Feature number = 7	17	816	566	785	56	65389	19	29891
Training samples = 1080 Feature number = 10	20	1147	632	794	59	66426	22	31880
Training samples = 1080 Feature number = 14	21	1626	658	1135	65	68562	24	34193

**Table 7:** Means and standard deviations (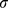) of overall classification accuracies based on various samples and features using ETM^+^ image.

Training condition	MLC		BPN		PSVM		FNPSVM	
	Mean	σ	Mean	σ	Mean	σ	Mean	σ
Training samples = 60 Testing samples = 210 Feature number = 4	80.62	1.2501	82.70	2.7309	85.62	2.6845	86.51	1.8390
Training samples = 60 Testing samples = 210 Feature number = 10	81.14	1.8666	82.76	3.6037	89.98	1.3334	90.29	1.0604
Training samples = 90 Testing samples = 180 Feature number = 4	82.05	1.2069	81.68	3.0933	86.18	2.4831	86.95	1.9367
Training samples = 90 Testing samples = 180 Feature number = 10	82.17	1.7292	83.61	2.8588	90.56	1.2268	90.54	0.9945
Training samples = 120 Testing samples = 150 Feature number = 4	80.83	1.1455	81.37	4.6063	84.36	2.4258	85.85	1.6640
Training samples = 120 Testing samples = 150 Feature number = 10	82.47	1.5977	84.50	3.3208	90.84	1.1358	91.74	1.0548

(1) Classification accuracy.In this paper, classification accuracy, one of the most important criterions in evaluating the performance of the classifier, was measured using overall accuracies and kappa coefficients computed by the confusion or error matrix. The most widely used way to represent the classification accuracy of remote sensing data should be in the form of an error matrix, applicable for a variety of site-specific accuracy assessments. Numerous researchers have recommended using error matrix in representing accuracy in the past, and it has now become one of the standard conventions to adopt such practice. The effectiveness of the error matrix in representing accuracy can be seen from the fact that accuracies of each category are fundamentally described along with both the errors of inclusion and errors of exclusion present in the classification [25], [45]. In order to accommodate the effects of chance agreement, some researchers suggest using kappa coefficient and adopting it as a standard measure of classification accuracy [46]. Foody [47]also pointed out that since many of the remote sensing data sets are dominated by mixed pixels, the standard accuracy assessment measures such as the kappa coefficient is often not suitable for accuracy assessment in remote sensing. Although its sensitivity to the density or frequency of the dynamic change in real world had some researchers arguing about its effect, the fact remains that the kappa coefficient has many intriguing features as an index of classification accuracy. More specifically, it offers some compensation for chance agreement, and a variance term could be calculated, enabling the statistical testing of the significance of the difference between two coefficients [25], [48].

We also need to emphasize that the various measures of accuracy are to evaluate different components of accuracy and to make different assumptions on the data [49]. The fact is that the measurement and meaning of classification accuracy depend substantially on individual perspective and demands [49], [50]. An accuracy assessment can be conducted for a variety of reasons, and many researchers have recommended that measures such as the kappa coefficient of agreement be adopted as a standard [25], [46].
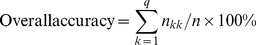


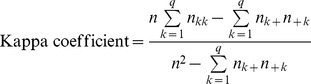
In terms of the above parameters selected from different algorithms, and basing on the 270 samples of each category obtained through simple random sampling design, we obtained overall classification accuracies and kappa coefficients using various multi-class strategies and classifiers on 12 training cases with the ETM^+^ dataset consisting of 4,037,099 points (see Table 5). Unfortunately, confronting such a large dataset, SVM failed on this problem because it required the more costly solution of a linear or quadratic program. Several patterns can be observed from Table 5 and Table 7, explained as follows:

1) As far as the multi-class classification strategies of PSVM and FNPSVM were concerned, the accuracies of “one-against-one” strategy in all training cases were about 1–2% higher than those of “one-against-rest” strategy. Also, through experiments, we found that compared to the classification speed of “one-against-rest” strategy, the classification speed of “one-against-one” strategy was at least two times higher, for both PSVM and FNPSVM (not listed in the following tables). So in this paper, we employed “one-against-one” multi-class classification strategy of PSVM and FNPSVM for comparison with the other two classifiers.

2) The level of classification accuracies achieved by PSVM and FNPSVM was significantly higher than that produced by either the MLC or BPN classifier. In addition, they yielded significantly better results than the MLC or BPN classifier did in all 12 training cases (Table 5). The accuracy differences between the PSVM and FNPSVM were rather small, and quite the same as that between the MLC and BPN (Table 5). The mean overall accuracies of the PSVM and FNPSVM were remarkably higher than those of MLC and BPN, however the differences between MLC and BPN or between PSVM and FNPSVM were only slight (Table 7). This is expected because the PSVM and FNPSVM are designed to locate an optimal separating hyperplane, while the other two algorithms may not be able to locate this separating hyperplane. Statistically, the optimal separating hyperplanes located by the PSVM and FNPSVM should be generalized to unseen samples with the least errors among all separating hyperplanes. Generally, as the number of available features increases, the overall accuracies and kappa coefficients of PSVM and FNPSVM grow gradually. Unexpectedly however, the increase in the number of available features didn’t always lead to an improvement of the accuracies of MLC and BP. On the contrary, MLC and BP showed better comparative performances on training cases with ten features than they did on training cases with fourteen features, which might be explained by the presence of a large number of irrelevant features that would hurt the classification performances. This again demonstrates the importance of feature selection. In terms of Table 5, it could be seen that the accuracies and kappa coefficients of the four algorithms improved with the increase in training data size, though not significantly.

3) The overall accuracy differences between MLC and BPN on the data set used in this study were generally small, and those between PSVM and FNPSVM were also not obvious. However, many of them were statistically significant.

(2) Training speed and classification speed.Training speed and classification speed are two important criterions in evaluating the performances of classification algorithms. Shown in Table 6, the training speed and classification speed of the four classifiers were substantially different. Generally, the training time and classification time rise with an increase in available features. The training speed of BPN was significantly lower than those of the other three classifiers because of its complex network structure. As far as classification speed was concerned, in all training cases, those of the PSVM and FNPSVM were remarkably lower than those of the MLC and BPN. The classification of the MLC and BPN in all training cases took from less than an hour to only a few minutes, while the PSVM and FNPSVM took more than several hours and ten hours, respectively. This was due to the fact that PSVM and FNPSVM involved large matrix calculation and reverse matrix operation during the process of classification. In addition, it should be noted that we have spent much time in searching for the optimal key parameters including the kernel parameters 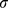 and the constant *c* in the training process, therefore yielding a better performance. Compared with PSVM, the training speed and classification speed of FNPSVM were more than twice its counterparts. The reason was that in terms of the comparison between the FNPSVM algorithm in section 2.2.1 and the PSVM algorithm [12], it was easy to find that the PSVM algorithm dealt with the product of an *n*-dimensional (*n* being the number of features) row vector and a matrix (*m* being the number of training samples), thus requiring a high calculation cost; while the FNPSVM algorithm avoided such problem.

To summarize, the training speeds and classification speeds of the above four algorithms are affected by many factors, including numbers of training samples and features, the size of training data set, as well as algorithm parameter settings. The training speed and classification speed of BPN depend on network structure, momentum rate, learning rate and converging criteria; while those of the PSVM and FNPSVM were affected by the number of features, kernel function, key parameter settings, as well as class separability.

(3) Algorithm stability.Various accuracies in Table 5 were obtained by randomly selecting training samples and testing samples only once at each sample size level. In order to evaluate the stabilities of the four classifiers and for the results to be statistically valid, we randomly selected training samples and testing samples for six times at three sample data size levels from the 270 samples of each class: 60 training samples and 210 testing samples, 90 training samples and 180 testing samples, as well as 120 training samples and 150 testing samples. Thus each classification algorithm was trained six times by various-sized training samples with four and ten features, respectively. Afterwards we calculated the means and standard deviations of the overall classification accuracies of each classifier (see Table 7).

**Figure 5:**
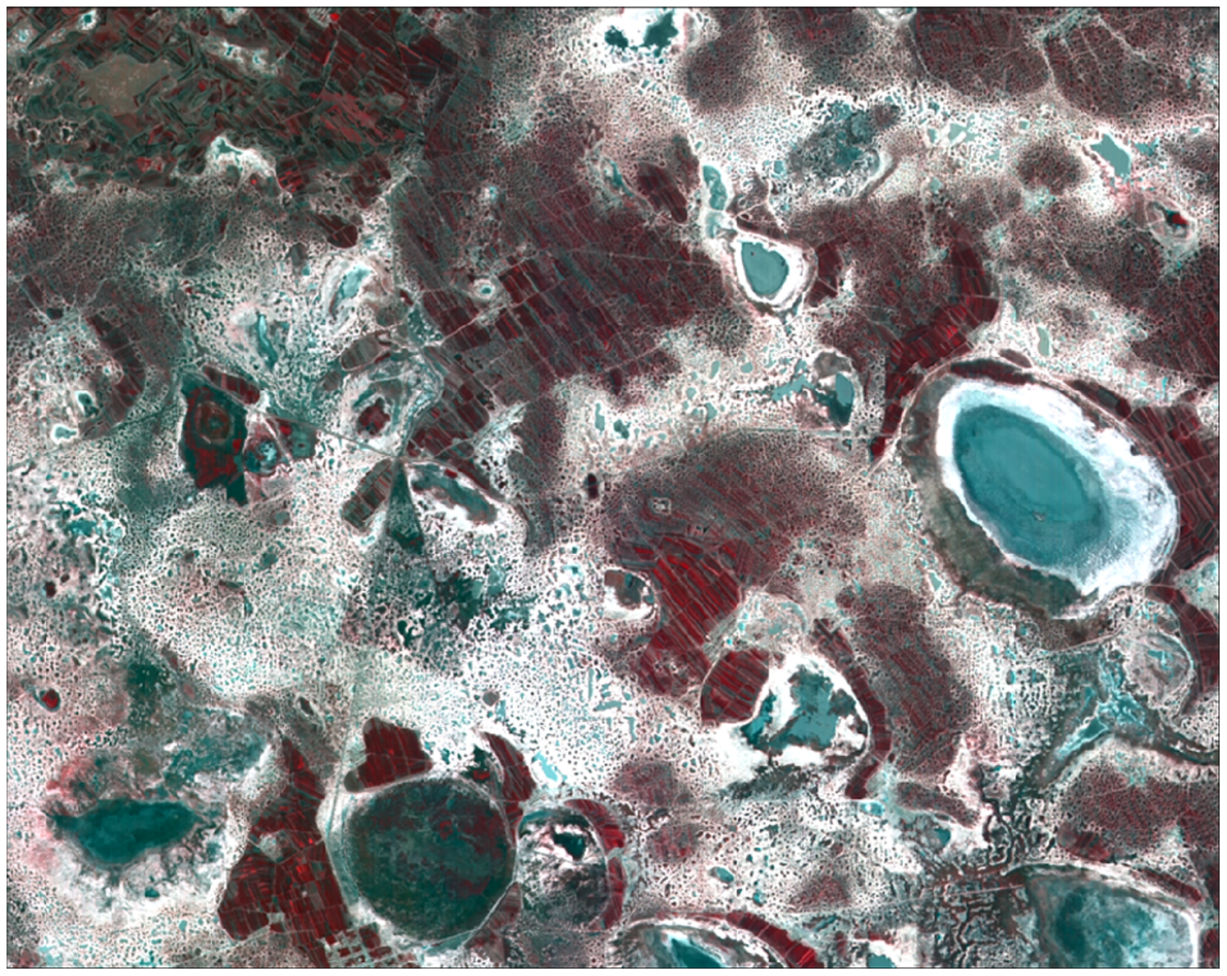
Original SPOT image in study area (composite of bands 3, 2 and 1).

The standard deviation of the overall accuracy of an algorithm estimated in cross validation is a quantitative measure of its relative stability [4]. Both Table 7 and Figure 4 revealed that the stabilities of the algorithms differed greatly and were affected by the training data size, testing data size, and the number of features. Generally, the overall classification accuracies of the algorithms became more robust when trained by using large-sized pixels than using small-sized pixels, especially when ten features were used (Figures 5 (*b*), (*d*), and (*f*)). Unexpectedly however, MLC showed higher reliability and lower mean overall accuracy when trained with only four out of a total 14 features (Table 7). This is probably due to the fact that MLC algorithm itself is sensitive to some relevant features, while some features that are partially or completely irrelevant to the classification target only increase the uncertainty of the classification results. On the other hand, according to Hughes effect [51], the effect of increasing dimensionality is thought to lower the reliability of the estimates of statistical parameters required for the computation of probabilities [10]. The FNPSVM showed more stable overall accuracies than the other three classifiers did when trained with ten features at different training sample size levels; however, when trained with four out of the total 14 features, the stability of FNPSVM was significantly lower than that of MLC, although clearly higher than those of the other two algorithms (Figure 4 (*a*), (*c*), and (*e*)). The likely cause of the stability of FNPSVM being lower than that of MLC on data with four features is that the applicability of the FNPSVM to non-linear decision boundaries depends on whether the decision boundaries can be transformed into linear ones by mapping the input data into a high-dimensional space. When the data contain very few features, the FNPSVM can’t successfully transform non-linear decision boundaries in the original feature space into linear ones in a high-dimensional feature space, while the MLC algorithm is useful when there is a fair amount of randomness under which the data are generated. The theoretical statistical distribution allows the use of the MLC approach that is optimal in the sense that, using too many irrelevant features probably affects its stability; so that even when the data contain very few features, it has better comparative reliability performance over the FNPSVM.

Compared to PSVM, FNPSVM generated better reliability in all of the 6 training cases (Table 7), owing to automatically associating each data point with a fuzzy membership that can reflect their relative degrees as meaningful data, and FNPSVM becoming more applicable in reducing the effects of noises or outliers in the process of training. Of the four algorithms, the BPN gave overall accuracies in a wider range than the other three algorithms (Figure 4) did for all cases, and showed the worst reliability (Table 7) because of its complex network structure and lots of optional parameters that affect the classification performance.

### Classification Experiments Using SPOT Image

#### Experiment summary

This study took the SPOT remote sensing image captured on September 12^th^, 2004 (scene number : 64002TH200409121049401018) as the data source, covering the western area of Da’an city in China and including near-infrared, red and green band. We cut 1,734*1,969 sized image from the SPOT image as test data set. The experiment area mainly contained several land types, namely heavy saline-alkalized land, moderate saline-alkalized land, light saline-alkalized land, water area and farmland. And then 120 samples (60 training samples and 60 test samples) were selected from each land type to train classification algorithm and to evaluate the accuracy of classification.

To evaluate the performance of FNPSVM algorithm on extracted saline-alkalized land using high spatial resolution (SPOT with 20m resolution), we adopted the “one-against-one” strategy based on the Gaussian RBF kernel function, and compared with MLC, BPN, and PSVM methods in terms of classification accuracy and classification speed.

#### Feature extraction

The paper extracted 8 features from the SPOT image data, including near-infrared band, red band, green band, the 1^st^ component of K-L transform, NDVI, and the H, S and I components of HSI color space. NDVI is expressed by the following formula:
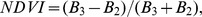
where 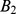 and 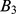 are red band and near-infrared band of SPOT, respectively. RGB image is acquired by basing on the false-color composite using the third, the second, and the first band of SPOT image. The RGB model is then transformed to HSI model by the following formulas:



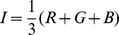


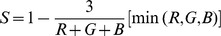


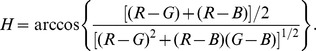



It’s not necessary to choose feature because of the limited features, so we extracted saline-alkalized land information based on the above eight features using various algorithms.

#### Key parameter setting

We used the method in section 3.4 to obtain the parameters of PSVM and FNPSVM classifier. The accuracy of FNPSVM classifier didn’t change significantly when 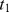 changed in the 0.05–0.2 range and 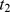 changed in the 0.7–0.9 range, so the paper still set 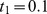 and 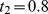. Afterwards, the optimal parameters of 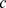 and 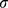 were searched, and the results were shown in Table 8 and 9. The overall classification accuracy increased to a maximum of 97.26% at 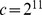 and 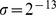.

**Table 8:** The overall accuracies (%) and kappa coefficients of the first layer grid-search using 6-time random-validation based on SPOT image.

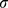 *c*	2^−14^	2^−10^	2^−6^	2^−2^	2^2^	2^6^	2^10^	2^14^
2^−14^	66.13/0.57	80.13/0.75	85.40/0.81	94.80/0.93	95.73/0.94	96.33/0.95	92.80/0.91	93.40/0.91
2^−10^	79.93/0.74	86.46/0.83	94.60/0.93	96.33/0.95	93.53/0.91	88.53/0.85	96.53/0.95	93.26/0.91
2^−6^	28.00/0.10	48.93/0.36	72.40/0.65	76.06/0.70	89.53/0.86	88.46/0.85	88.93/0.86	84.73/0.81
2^−2^	20.00/0	20.86/0.01	24.86/0.06	42.60/0.28	40.53/0.25	44.26/0.30	45.40/0.31	48.73/0.36
2^2^	20.00/0	20.06/0	20.40/0	22.33/0.03	24.06/0.05	24.53/0.05	23.80/0.04	24.06/0.05
2^6^	20.00/0	20.20/0	20.33/0	20.93/0.01	21.20/0.01	21.46/0.02	20.80/0.01	21.26/0.01
2^10^	20.00/0	20.00/0	20.06/0	20.33/0	20.20/0	20.60/0.01	20.53/0.01	20.60/0.01
2^14^	20.00/0	20.13/0	20.26/0	20.46/0	20.73/0.01	20.66/0.01	20.60/0.01	20.33/0

**Table 9:** The overall accuracies (%) and kappa coefficients of the second layer grid-search using 6-time random-validation based on SPOT image.

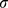 *c*	2^7^	2^8^	2^9^	2^10^	2^11^	2^12^	2^13^
2^−7^	94.33/0.93	92.46/0.91	91.86/0.89	94.53/0.93	87.73/0.84	83.66/0.79	91.60/0.89
2^−8^	92.33/0.90	96.46/0.95	92.00/0.90	91.66/0.89	91.60/0.89	92.00/0.90	91.86/0.90
2^−9^	97.60/0.97	93.20/0.91	93.13/0.91	96.73/0.95	92.73/0.91	96.6/0.95	96.53/0.95
2^−10^	96.33/0.95	96.46/0.95	97.60/0.97	97.13/0.96	89.53/0.87	92.86/0.91	96.60/0.95
2^−11^	92.80/0.91	92.46/0.91	97.06/0.96	97.00/0.96	96.86/0.96	97.13/0.96	96.73/0.96
2^−12^	96.86/0.96	96.53/0.95	97.00/0.96	89.13/0.86	93.73/0.92	96.66/0.95	97.00/0.96
2^−13^	93.00/0.91	96.86/0.96	89.66/0.87	96.73/0.95	97.26/0.96	88.46/0.85	92.33/0.90

The parameter setting of BPN was as same as the BPN parameters of section 4.1.3.2 except for the neural network structure. We chose log-sigmoid function as the transfer functions from input layer to output layer, and set the limit on the neural network’s iteration number to 1,000 times for each desired output. Levenberg-Marquard optimum algorithm is used as the training function. The other parameters of the network were set as follows: learning rate 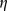 = 0.3, momentum factor 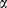 = 0.8, minimum gradient 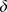 = 10–20, and minimum mean square error 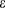 = 10-6. In this study, the number of neurons in the hidden layer is finally acquired through repeated experiments, eventually arriving at 12. Therefore the structure of neural network is 8-12-5.

#### Performance assessments

**Figure 6:**
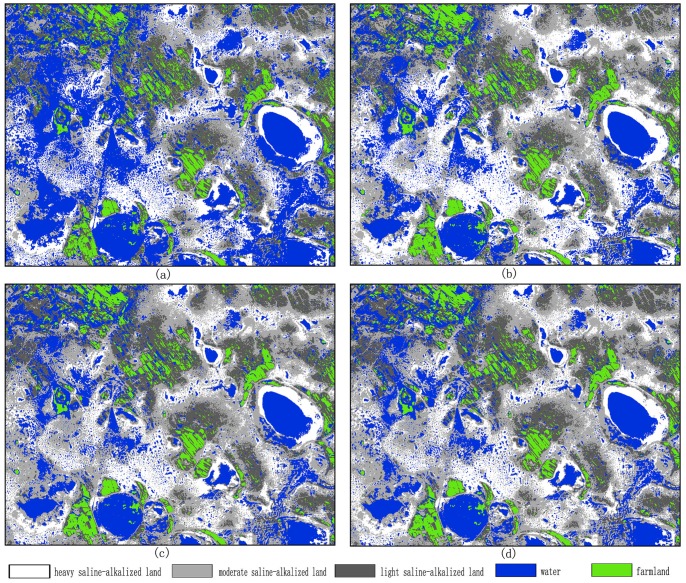
Classification maps for the western part of test area of Da’an city in China using various classifiers under the same training cases (120 training samples for each class, 8 features) based on SPOT image. (a) MLC algorithm. (b) BPN algorithm, 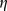 = 0.3, 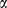 = 0.8, 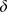 = 10^−20^, 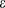 = 10^−6^. (c) PSVM algorithm, c = 2^11^, 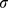 = 2^−13^. (d) FNPSVM, t1 = 0.1, t2 = 0.8, c = 2^11^ and 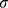 = 2^−13^.

(1) Vision effect.Original SPOT image in study area is obtained by compositing bands 3, 2 and 1 (see Figure 5). Figure 6 shows the experiment classification result using MLC, BPN, PSVM and FNPSVM algorithms which were based on 8 feature vectors and 60 training samples per class. When comparing the classification result map of each algorithm with the original SPOT image, on the macro level the differences of various classification result maps are not clear; but in detail we can find that the spatial patterns of land cover classification from the PSVM and FNPSVM method are significantly better than the other two methods, while the spatial patterns of classification maps are quite similar between PSVM and FNPSVM. As seen in Figure 6, when using MLC and BPN classifier, not only were the patches fragmented, but the saline-alkalized land and water area were also mistakenly mixed; and a large number of saline-alkalized lands were wrongly classified as water area. But BPN, PSVM and FNPSVM classifiers all overcome the drawbacks of MLC classifier.

(2) Classification accuracy.We obtained the confusion matrix according to 60 test samples, and then calculated the overall accuracy and kappa coefficient of various classifiers (see Table 10).

**Table 10:** Overall accuracies (%), kappa coefficients and classification speed using various classifiers based on SPOT image.

	*MLC*	*BPN*	*PSVM*	*FNPSVM*
overall accuracy	89.67	95.33	96.33	97.33
kappacoefficients	0.87	0.94	0.95	0.97
classificationspeed	153	407	17167	8285

Seen from Table 10, the overall accuracy and kappa coefficient of all classifiers were higher, except that the classification accuracy of MLC classifier was less than 90%. The accuracies of the other classifiers were all higher than 95%. The overall accuracy of FNPSVM was the highest (97.33%), meaning that the performance of FNPSVM was better than the others, which was mainly in accordance to the strict mathematical theory.

(3) Classification speed.Classification speed is one of the important indicators to evaluate the performance of the classifier. Table 10 gave the classification time based on the 8-dimensional feature vector. As can be seen from the table, the MLC classifier was the fastest, mainly because of the simplicity of the algorithm and the low amount of computation. This was followed by BPN classification by only a few minutes. The classification speed of PSVM and FNPSVM dropped substantially from that, with several hours lagging; the speed of FNPSVM was twice the such of PSVM, for the same reason that explains ETM image classification speed, of which the paper will not discuss at this time.

(4) Algorithm stability.Based on the above experiment data, feature extraction and key parameter settings, we obtained the overall classification accuracies of various classifiers (see Table 10), and calculated the means and standard deviations (see Table 11).

**Table 11:** Means and standard deviations (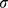) of overall classification accuracies based on SPOT image.

	MLC	BPN	PSVM	FNPSVM
Mean	81.43	82.66	89.72	90.79
σ	1.9212	3.5050	1.3646	1.0211

Seen from Table 11, the stability of each algorithm under the same training condition is quite different. The stability of BPN classifier is the lowest because of its complex network structure and the many optional parameters affecting the classification performance. The FNPSVM showed more stable overall accuracies than the other three classifiers did, as by automatically associating each data point with a fuzzy membership in the process of training, FNPSVM could effectively reduce the effects of noises.

### Conclusions

Considered as a kind of regularized least squares SVM, PSVM requires the solution of a single set of linear equations, and thus can be considerably faster than conventional SVM. Jayadeva [16] extended the PSVM and proposed fuzzy linear proximal support vector machine. In order to increase nonlinear separability of real data set, we presented in this paper the fuzzy nonlinear proximal support vector machine (FNPSVM), and described the strategy for setting fuzzy membership in FNPSVM, therefore making FNPSVM more feasible in the application of reducing the effects of noises or outliers. Numerous experiments were performed to evaluate the comparative performances of this algorithm and three other popular classifiers, including the MLC, BPN and PSVM in saline-alkalized land classification. In addition, impacts of the key parameters of FNPSVM algorithm on its performance as well as the impacts of the selection of training data and features on all four classifiers were also evaluated.

The results of our experiments supported the use of “one-against-one” strategy for multi-class classification problems, and indicated that of the four algorithms evaluated, both the PSVM and FNPSVM achieved considerably higher levels in overall accuracies and kappa coefficients than either the MLC or the BPN did, especially so in high-dimensional feature space; and comparatively, the accuracies and kappa coefficients of the MLC were lowest in all 12 training cases. The results should be attributed to the abilities of PSVM and FNPSVM in locating the optimal separating hyperplanes, as shown in Figure 1. Statistically, the optimal separating hyperplanes found by the PSVM and FNPSVM classifiers should be generalized as unlabeled samples with errors smaller than any other separating hyperplanes that might be located by other classifiers. In terms of the performances of PSVM and FNPSVM classifiers, the absolute differences of their overall accuracies were quite small. Many of the differences were however, statistically significant.

The stabilities of PSVM and FNPSVM are closely correlated to the features used in the classification. The PSVM and FNPSVM algorithms gave higher stability than either the MLC or BPN did when being trained with 10 features by different sizes of pixels. When reduced to only 4 features however, the MLC manifested comparatively better reliability. As far as the PSVM and FNPSVM classifiers were concerned, the stability of FNPSVM was significantly higher than that of the PSVM, because the application of fuzzy set approach reduced the effects of noises or outliers. With regard to classification speed, based on larger dataset consisting of 4,037,099 points, the MLC and BPN were much faster than the PSVM and FNPSVM, and the computational cost of the PSVM was more than twice the cost of the FNPSVM. This indicated that the algorithm in this paper had predominant advantage in running speed compared to PSVM, and noted that both the PSVM and FNPSVM were affected by training data size, key parameter settings, class separability and so on.

When we adopted the multi-layer grid search and random-validation method in searching for the optimal parameters 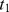 and 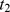, it was found that with 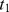 valued smaller than 0.2 and 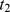 valued larger than 0.7, accuracy variation of FNPSVM classifier was rather small, and the classification accuracy was relatively high. Within this range, the sensitivity wasn’t high. With 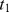 valued between 0.2 and 0.5, or 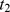 valued between 0.5 and 0.7, accuracy variation of FNPSVM classifier was relatively high, while the classification accuracy significantly reduced, showing obvious cases of misclassification. Within this range, the sensitivity was relatively high. This is mainly because 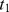 and 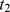 would determine the range of which this sample point absolutely does or doesn’t belong to a given class. When 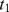 has too high a value or 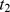 too low a value, it would automatically lead to a higher probability in misclassification, at which point the classification result is relatively sensitive to the values of 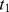 and 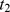. This is also in consistency with our experience.

Both the selection and the number of training samples and testing samples affect the performances of all four classifiers. It is impractical and even impossible to determine the minimum number of samples needed for the sufficient training of an algorithm according to the results from these experiments. Fuzzy classification technique can reduce the corruption that the noises have on the data samples, and consequently, classification robustness is improved. To a greater extent, feature extraction and feature selection exert a strong impact on the substantial increases in accuracy, as some irrelevant features probably lead to a declining classification performance for some algorithms. As SVM is becoming a popular learning machine for object classification, the principal contribution of this paper is the presentation of a fast, simple and efficient classification algorithm in the research field of SVM, which is important as it significantly reduces the running time of classification and improves the stability performance.

Currently, due to China’s increasing population and the far from perfect land management system, the conflict in more people having less land is becoming increasingly apparent. Drastically reduced farmland and land degradation have drawn the public’s attention. The FNPSVM method proposed in this paper can rapidly and accurately extract information in land use and dynamic changes. As a foundation in establishing general planning for land utilization, protecting basic farmland, and ensuring sustainable development of the land, the method is also capable of providing decision supports for the authorities in land utilization and management. The algorithm has been successfully used in lands extraction with remote sensing image, and with the development in constructing the digital city, the extraction of land use information in cities becomes increasingly important. This algorithm can be used to classify patches of cities, and it is devoted to the construction of a digital city.
